# Effect of Jasmonic Acid Foliar Spray on the Morpho-Physiological Mechanism of Salt Stress Tolerance in Two Soybean Varieties (*Glycine max* L.)

**DOI:** 10.3390/plants11050651

**Published:** 2022-02-28

**Authors:** Javaria Noor, Abd Ullah, Muhammad Hamzah Saleem, Akash Tariq, Sami Ullah, Abdul Waheed, Mohammad K. Okla, Abdulrahman Al-Hashimi, Yinglong Chen, Zeeshan Ahmed, Izhar Ahmad

**Affiliations:** 1 Department of Botany, Islamia College University, Peshawar 25120, Pakistan; jnoorbotanist@gmail.com; 2 Xinjiang Key Laboratory of Desert Plant Roots Ecology and Vegetation Restoration, Xinjiang Institute of Ecology and Geography, Chinese Academy of Sciences, Urumqi 830011, China; abdullahbotany123@gmail.com; 3 State Key Laboratory of Desert and Oasis Ecology, Xinjiang Institute of Ecology and Geography, Chinese Academy of Sciences, Urumqi 830011, China; waheedfafu@gmail.com (A.W.); zeeshanagronomist@yahoo.com (Z.A.); 4 Cele National Station of Observation and Research for Desert-Grassland Ecosystems, Cele 848300, China; 5 College of Plant Science and Technology, Huazhong Agricultural University, Wuhan 430070, China; saleemhamza312@webmail.hzau.edu.cn; 6 Department of Botany, University of Peshawar, Peshawar 25120, Pakistan; samibotany@uop.edu.pk; 7 Department of Botany and Microbiology, College of Science, King Saud University, Riyadh 11451, Saudi Arabia; malokla@ksu.edu.sa (M.K.O.); aalhashimi@ksu.edu.sa (A.A.-H.); 8 The UWA Institute of Agriculture, UWA School of Agriculture and Environment, The University of Western Australia, Perth, WA 6001, Australia; yinglong.chen@uwa.edu.au

**Keywords:** salinity, photosynthesis, soluble protein, phenol, ion’s homeostasis

## Abstract

Jasmonates (JAs) are lipid-derived compounds that function in plants as key signaling compounds during stressful conditions. This study aimed to examine the effects of exogenous fo-liar-JA application (100 μmol L^−1^) on the morpho-physiological response of two soybean varieties (parachinar-local and swat-84) grown under different NaCl regimes (0, 40, 80, and 120 mM). Results show that exogenous JA application alone and in combination with salt stress altered the growth and metabolism of both soybeans. For instance, they accumulated significant amounts of Na^+^ and Cl^–^, while their K^+^, Mg^2+^, Fe^2+^, Mn^2+^, B^3+^, and P^3+^ contents were low. Further, photosynthetic pigments Chl a and Chl b increased at low concentrations of salt and exogenous JA. Car decreased under both salt and exogenous JA as compared with untreated control. In addition, sugar, phenol, and protein content increased under both salt and exogenous JA application. In contrast, the exogenous JA application alleviated the negative impact of salt stress on the growth and metabolism of both soybeans. Further, the high concentrations of soluble protein and phenol in the leaves of both soybeans may contribute to their ability to adapt to salinity. However, molecular studies are necessary to understand the ameliorative role of exogenous JA in the growth and metabolism of salt-treated young seedlings in both soybean varieties.

## 1. Introduction

Salinity is one of the most significant abiotic stress factors that negatively impact crop growth, productivity, and distribution worldwide [1,2]. Salinity affects approximately 20% (45 million ha) of irrigated land, which produces one-third of the world’s food. As a result of more salt-affected areas, 20% of agricultural areas are expected to disappear within the next 25 years [3]. The detrimental effects of salt stress can primarily be attributed to high Na^+^ and Cl^–^ concentrations. Salinity can lead to ionic toxicity, diminished nutrient uptake, metabolic toxicity, membrane disorganization, photosynthesis inhibition, and oxidative damage [4,5].

The process of photosynthesis is sensitive to environmental factors [6]. For instance, NaCl stress severely damages the receptor side of photosystem II of soybean leaves and disrupts the normal photosynthesis process [7]. The reduction or inhibition of photosynthetic activity due to salt stress is primarily caused by toxic accumulations of Na^+^ and Cl^−^ [8]. Additionally, the uptake and homeostasis of mineral ions are critical for plants’ normal development and growth [9,10]. In addition to causing ion toxicity in plants, excessive salt stress also affects the uptake of important mineral ions such as K^+^, Mg^2+^, Fe^2+^, Mn^2+^, Zn^2+^, and B^3+^ [11,12]. Furthermore, salt stress results in the overproduction of reactive oxygen species (ROS), which damage cell membranes, proteins, DNA, lipids, and other metabolic components [13]. 

Plants are adapted to survive under stress conditions by arresting their development and prioritizing defense mechanisms. This causes physiological and developmental changes in the plant. In all of these processes, hormones play a critical role [14,15]. It has been demonstrated that the biosynthesis and accumulation of phytohormones such as abscisic acid [16], salicylic acid [17], and jasmonate [18,19] form a pivotal adaptive strategy of plants to abiotic stresses. Hence, the application of phytohormones has been considered an effective way to improve plant growth and mitigate the effects of salt stress [20]. Jasmonic acid (JA) and growth-related hormones antagonistically interact to coordinate plant growth and defense [14]. Both methyl jasmonate (MeJA) and jasmonic acid (JA), collectively referred to as jasmonates, are considered to have a beneficial effect on various crops subjected to salt stress [21]. For instance, exogenous MeJA has been reported to ameliorate the adverse effects of salt stress on chlorophyll concentration, photosynthetic rate, transpiration rate, proline content, and overall growth of plants [21]. Additionally, the application of exogenous JA can enhance stress tolerance by improving the antioxidant system, which includes enzymes and metabolites to scavenge the excessive ROSs generated as a result of various abiotic shocks [22,23]. 

Furthermore, exogenous application of MeJA increases the accumulation of osmolytes, such as proline, ABA, and soluble sugars, thus facilitating plants’ adaptation to various abiotic stresses [24,25,26,27,28,29]. JA also plays a crucial role in maintaining ionic homeostasis. For instance, JA application has been reported to reduce the Na^+^/K^+^ ratios in salt-stressed maize seedlings and hence decrease the problem of ionic toxicity and ameliorate the effects of alkaline stress on maize roots and leaves [30]. In addition, in salt-sensitive rice seedlings exposed to salt stress, exogenous MeJA decreased the uptake of Na^+^ but enhanced the uptake of Mg^2+^, Ca^2+^, and K^+^ [31]. Therefore, crosstalk with photosynthesis, anti-oxidants, osmolytes, and ionic homeostasis are important mechanisms by which JAs improve salt tolerance in plants. However, this is highly dependent upon the plant species, the type and intensity of stress, growth stage, and concentration of JA application. 

Cultivated soybean (*Glycine max* L.) belongs to the plant family Leguminosae. It is a rich source of protein and oil for humans and animals [32]. It is generally a salt-sensitive species and requires genetic improvement to thrive in salinized soils [33]. Since salt-affected lands are increasing day by day, which will reduce cultivated lands in the near future [3], it is critical to evaluate traits related to salt tolerance in plants to develop crops that will survive in salt-affected soils [34]. To the best of our knowledge, the application of exogenous JA-foliar to salt-grown parachinar-local and swat-84 soybean varieties has not been evaluated for its possible ameliorative effect. We, therefore, conducted this study (a) to evaluate and compare the changes in growth, photosynthetic ability, ion accumulation, and important biochemical indicators in parachinar-local and swat-84 soybean under various NaCl stress conditions and exogenous JA application, (b) to understand the physiological mechanisms of salt tolerance, and (c) to develop salt-tolerant soybean varieties on a scientific basis.

## 2. Results

### 2.1. Changes in Seedlings Growth

We observed that salt stress and exogenous jasmonic acid (JA) application altered the growth characteristics of both soybean varieties (Table 1 and Table 2). For instance, the lowest shoot height, shoot fresh weight, and moisture content of both soybeans were observed at a high salt concentration (T7, 120 mM NaCl). In addition, shoot height of parachinar-local soybean increased at exogenous JA application (T2, jasmonic acid) and low salt alone and with JA application (T3, 40 mM NaCl + JA). In addition, the highest shoot fresh weight of parachinar-local soybean was recorded at high salt with exogenous JA application among the treatments (Table 1). Additionally, the presence of high salt concentration also greatly reduced the root length and fresh weight of both types of soybeans (Table 2). Among the treatment groups, roots length, root fresh weight, and moisture content of swat-84 soybean increased at T8 (120 mM NaCl + JA), while those of parachinar-local soybean increased at T6 (80 mM NaCl + JA). Therefore, our results indicate that exogenous JA foliar spray has a positive effect by enhancing the biomass of plants subjected to NaCl stress.

### 2.2. Changes in Photosynthetic Pigments

**Figure 1:**
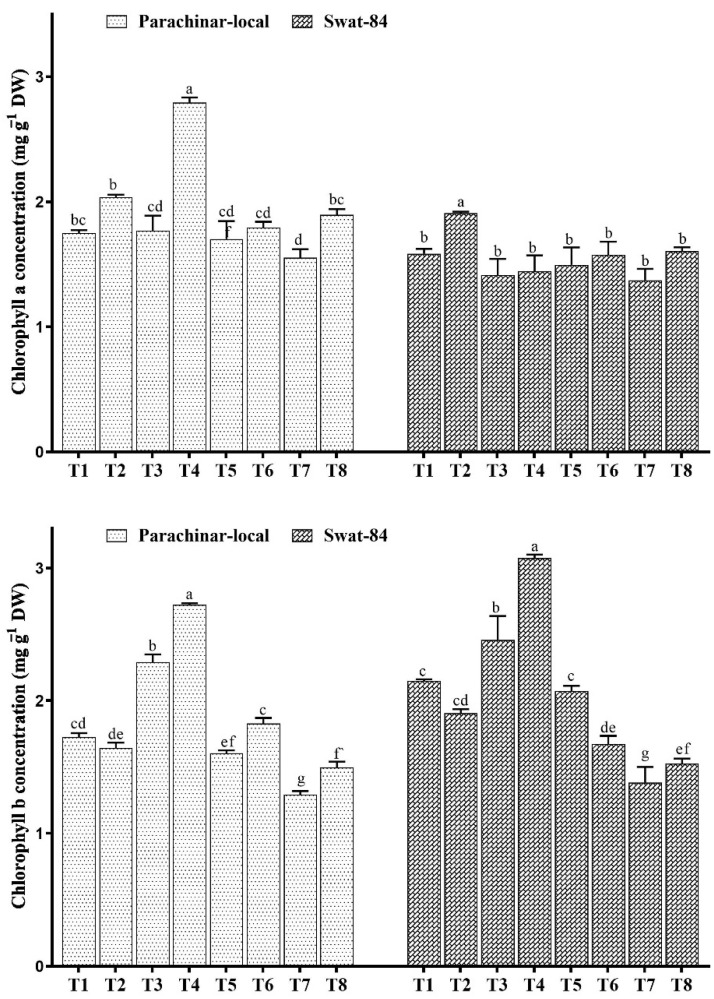
Changes in photosynthetic pigments under salt stress and exogenous JA application. Different letters for each column for each variety show significantly different values at *p* < 0.05 according to Duncan’s method. T1 (Control), T2 (Control + JA), T3 (40 mM), T4 (40 mM + JA), T5 (80 mM), T6 (80 mM + JA), T7 (120 mM), T8 (120 mM + JA).

**Figure 2:**
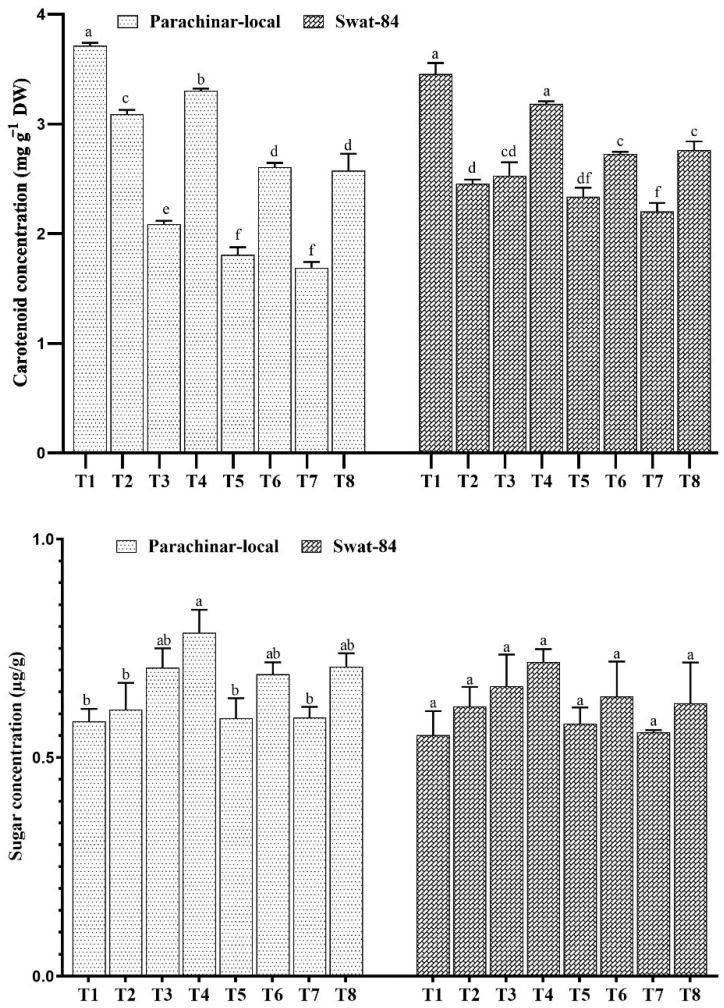
Changes in carotenoids and sugar concentration under salt stress and exogenous JA application. Different letters for each column for each variety show significantly different values at *p* < 0.05 according to Duncan’s method. T1 (Control), T2 (Control + JA), T3 (40 mM), T4 (40 mM + JA), T5 (80 mM), T6 (80 mM + JA), T7 (120 mM), T8 (120 mM + JA).

The results show that the photosynthetic capacities of the two soybean species differ in response to salt and exogenous JA application. High concentrations of photosynthetic pigments chlorophyll a (Chl a) and chlorophyll b (Chl b) were observed in both varieties under low salt stress (40 mM NaCl) and exogenous JA spray (Figure 1), whereas low concentrations were observed under high salt stress in comparison with control (Figure 1 and Figure 2). Furthermore, both salt and exogenous JA treatment reduced the Car pigment in both soybeans in comparison to the control. In contrast, exogenous JA foliar application in combination with salt stress increased the photosynthetic pigments Chl a, Chl b, and Car in both soybeans (Figure 1 and Figure 2). 

### 2.3. Changes in Foliar Ions Accumulation

The anion accumulation in leaves of both soybeans showed clear variations in response to salt stress and exogenous JA foliar spray (Table 3, Table 4 and Table 5). The concentrations of Na^+^ and Cl^−^ ions increased in both soybeans as the NaCl stress increased. In contrast, exogenous JA application combined with salt stress (T6, T8; 80 mM and 120 mM NaCl + JA) reduced Na^+^ and Cl^−^ accumulations (Table 3 and Table 4). Moreover, high Na^+^ and Cl^−^ ions severely reduced the concentration of K^+^, NO_3_^−^, Fe^3+^, Mg^2+^, Mn^2+^, B^3+^, P^3+^, NO_3_^−^, Zn^2+^, and H_2_PO_4_^−^ ions in leaves of both soybeans. Exogenous JA sprays, as well as NaCl stress, reduced NO_3_^−^ and H2PO_4_^−^ accumulation in all concentrations (Table 4 and Table 5). In the treatment group, higher Zn^3+^ and Mg^2+^ contents were observed with exogenous JA in both soybean varieties. In comparison to the control, Mn^2+^ content in swat-84 soybean and B^3+^ content in both soybeans decreased. Exogenous JA application, however, increased their concentrations in combination with salt stress (Table 5). Additionally, the Fe^2+^ content of soybean leaves increased with increasing salt stress as well as exogenous JA application in comparison with controls; maximum Fe^2+^ accumulation was observed at high salt stress and exogenous JA application in both soybean leaves (Table 5). 

### 2.4. Changes in Foliar Sugar, Protein, Phenol, and Vitamin A Concentration 

**Figure 3:**
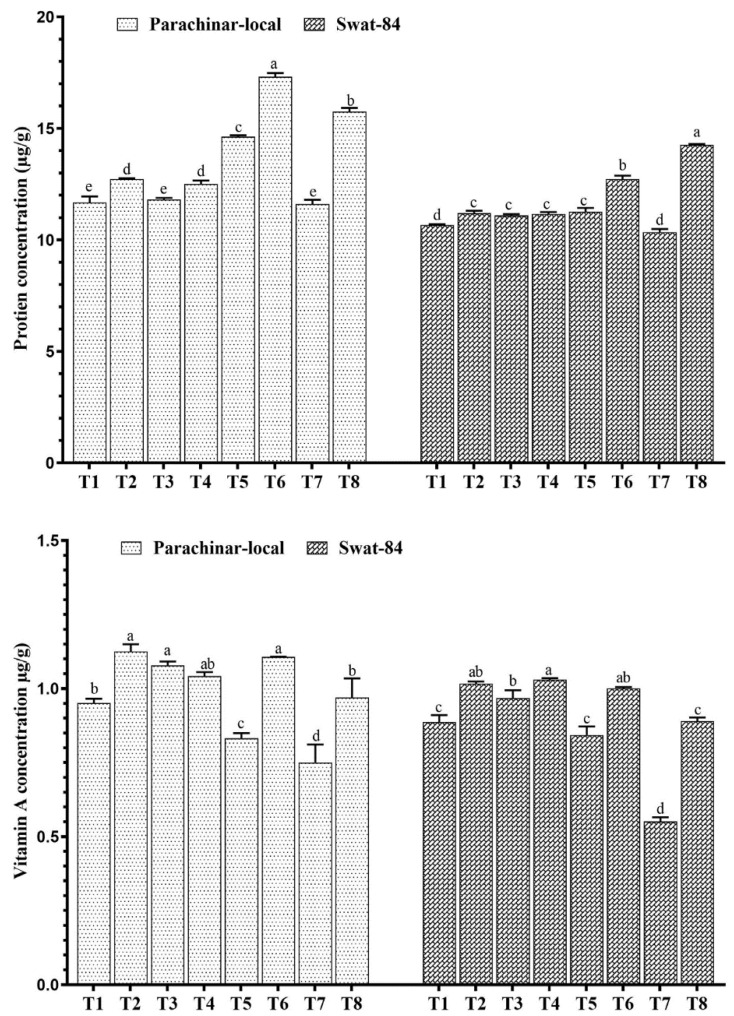
Changes in protein and vitamin A concentration under salt stress and exogenous JA application. Different letters for each column for each variety show significantly different values at *p* < 0.05 according to Duncan’s method. T1 (Control), T2 (Control + JA), T3 (40 mM), T4 (40 mM + JA), T5 (80 mM), T6 (80 mM + JA), T7 (120 mM), T8 (120 mM + JA).

Sugar levels in both soybeans increased under NaCl stress and exogenous JA foliar application, compared with that in the control. Sugar content was maximum under low salt stress (40 mM NaCl) and exogenous JA application in both soybean varieties (Figure 2). Moreover, the protein content in both soybean varieties increased in medium salt conditions and decreased in high salt conditions. In contrast, exogenous JA application increased protein content in parachinar-local soybeans at T6 (80 mM NaCl + JA), followed by T8 (120 mM + JA), as well as in swat-84 under medium salt stress (Figure 3). 

**Figure 4:**
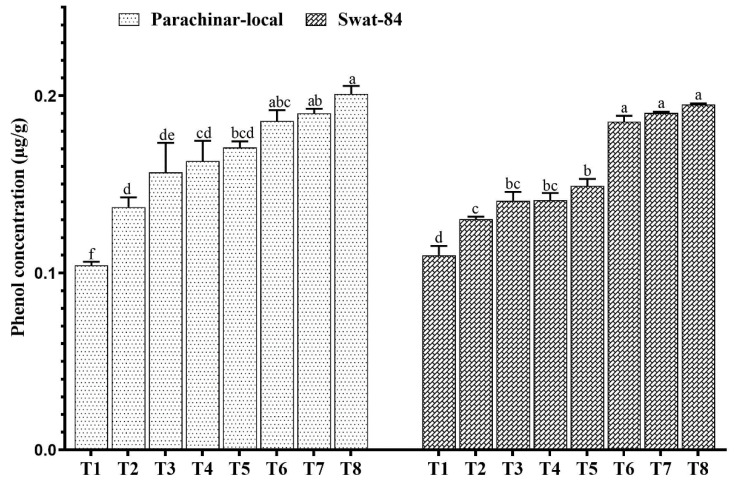
Changes in phenol concentration under salt stress and exogenous JA application. Different letters for each column for each variety show significantly different values at *p* < 0.05 according to Duncan’s method. T1 (Control), T2 (Control + JA), T3 (40 mM), T4 (40 mM + JA), T5 (80 mM), T6 (80 mM + JA), T7 (120 mM), T8 (120 mM + JA).

In addition, the vitamin A concentration increased at low salt concentrations (40 mM NaCl) but decreased at medium (80 mM NaCl) and high (120 mM NaCl) salt concentrations. When compared with that in the control, the highest vitamin A concentration was observed at exogenous JA application, followed by medium salt (80 mM NaCl) with exogenous JA application in parachinar-local, whereas, in swat-84, the highest vitamin A concentration was observed at low salt (40 mM NaCl) and exogenous JA application (Figure 3). Additionally, the phenol concentration increased with increasing salt stress alone and in combination with exogenous JA application in both soybeans (Figure 4). There was, however, a greater increase in phenol in plants exposed to salt and exogenous JA spray combination. Sugar, phenol, protein, and vitamin A concentrations of the parachinar-local variety were much higher than those of the swat-84 variety.

## 3. Discussion

Salinity is a major abiotic stress factor that adversely affects seed germination, plant growth, and productivity [35,36,37]. The phytohormone jasmonic acid (JA) can influence plant growth and development and regulates plant responses to biotic and abiotic factors [23,38,39,40,41,42,43].

This study examined the effect of exogenous JA application under four salt conditions (0, 40, 80, and 120 mM NaCl) on two soybean varieties, parachinar-local and swat-84, to determine its beneficial effects based on morphological features and physio-biochemical processes. While salt concentration simulations resulted in a reduction in the growth characteristics of both varieties of soybean, exogenous JA application had a significant impact on the shoot and root growth. Among the treatment groups, roots length, fresh weight, and dry weight of swat-84 soybean increased at high salt and exogenous JA application, while those of parachinar-local soybean increased at medium salt and exogenous JA application. Therefore, exogenous JA-foliar spray had a positive effect by promoting root growth [44,45,46]. A recent study demonstrated that overexpression of proteins that represent Protein NINJA homolog1 or Jasmonate ZIM domain (JAZ) proteins with mutations, deletions, or variations in the JA domain could suppress the inhibition of root growth caused by exogenous application of JA [46]. Although this might apply to our study, further validation is required.

In addition, low salt and exogenous JA application increased shoot height and fresh and dry weight of parachinar-local insignificantly. Low-medium salt in parachinar local and all concentrations of salt in swat-84 reduced the shoot growth parameters alone and in combination with the exogenous foliar-JA application. In a recent study, it was demonstrated that exogenous MeJA inhibited the growth of seedlings of *Nitraria tangutorum*, resulting in shorter shoots, smaller internodes, and lower fresh weight [47]. Plants that are exposed to environmental stresses experience growth–defense tradeoffs, and JA is thought to be a key factor in these tradeoffs [14,15].

Under conditions of salt stress, photosynthetic efficiency may decrease due to a lack of water available to the plants as a result of the surrounding low water potential [48]. The reduction of photosynthesis under salinity stress might be due to the deficit of water availability to the plants under salinity stress. In the present study, the concentrations of photosynthetic pigments Chl a and Chl b of both soybeans decreased with increasing salt stress. The decrease in photosynthetic pigments under salt stress has been observed in different plant species [11,49,50,51]. Among the treatment groups, exogenous JA application with salt stress improved photosynthetic pigments in comparison with salt stress alone. These findings are supported by previous studies, indicating that exogenous JA application can improve the photosynthetic pigments under salinity conditions [21,37,39,44,52,53]. Possibly, the increased content of chlorophyll pigments was caused by the improved activity of enzymes that are involved in the biosynthesis of chlorophyll, such as protochlorophyllide reductase and α-aminolevulinic acid dehydratase [54,55,56]. 

Plants produce chlorophyll through two major enzymes, namely, α-aminolevulinic acid dehydratase and protochlorophyllide reductase [56,57]. Protochlorophyllide reductase is known for enhancing the chlorophyll content of higher plants by reducing protochlorophyllide under the influence of light [50,55]. Furthermore, the enzyme α -aminolevulinic acid dehydratase is a common precursor of the tetrapyrrole ring in the chlorophyll structure, which contributes to alleviating some stress conditions by enhancing chlorophyll synthesis [54]. Moreover, both salt and exogenous JA reduced the performance of the Car pigment in comparison with the control, for instance, displaying a decreasing trend with an increasing salt concentration in both soybeans. However, exogenous JA foliar application improved the Car contents in salt-subjected seedlings of both soybeans, which are in line with previous findings [44,58]. In our study, sugar concentration increased under both salt stress and exogenous JA foliar application. parachinar-local soybean accumulated much sugar content in comparison to swat-84 under salt stress, which is considered beneficial for plant salt stress acclimation [11,52,59,60]. Plant metabolism is normalized through the use of this adaptive strategy, facilitating protein turnover and compatible solute production [60]. Soluble sugar adjusts the osmotic potential and enables better water absorption in salinity [61,62]. 

In the present study, Na^+^ and Cl^−^ ions increased in salt-exposed soybean seedlings of both varieties. The increase in Na^+^ and Cl^−^ accumulation due to salt can result in oxidative stress, which adversely affects plant growth and metabolism [11,63,64]. The present study demonstrated that salt stress reduced the accumulation of K^+^, Fe^2+^, Mg^2+^, Mn^2+^, B^3+^, and P^3+^ ions in the leaves of both soybean varieties. The toxic accumulations of Na^+^ and Cl^−^ ions can reduce the uptake and accumulation of important mineral elements [11,65,66]. The imbalances in inorganic ions severely restrict plant growth and productivity by hindering osmotic adjustment and turgor maintenance, photosynthesis, nitrogen assimilation, and protein synthesis [4,11,23,67]. In contrast, exogenous JA application reduced the accumulation of both toxic Na^+^ and Cl^−^ ions in salt-treated seedlings of both soybean types. Our results are in line with previous studies claiming that exogenous JA application plays a key role in maintaining ionic homeostasis [30,38,43]. For instance, consider the increase in accumulation of Na^+^ and the decrease in K^+^ ion uptake. 

It has been reported that salt stress decreases Na^+^/K^+^ ratios in salt-stressed maize seedlings, reducing ionic toxicity and ameliorating the effects of alkaline stress on maize roots and leaves [30]. In another study, in salt-sensitive rice seedlings exposed to salt stress, the exogenous application of MeJA decreased the uptake of Na^+^ but enhanced the uptake of Mg^2+^, Ca^2+^, and K^+^ [31]. Additionally, exogenous JA application alone and in combination with salt stress increased the concentration of H_2_PO_4_^−^ in both soybean varieties. Under salt stress, the plant needs to accumulate high amounts of H_2_PO_4_^−^ to counter the massive Na_+_ influx [11,68].

In the present study, the concentration of NO_3_^−^ decreased with increasing salt stress. Reductions in the uptake and accumulation of NO_3_^−^ may result in a greater reduction in the dry weight of a plant [69]. Additionally, proteins play an important role in salt tolerance, acclimatization, and cellular adjustment in plants. The present study found that, in both soybean varieties, protein increased at low and medium salt concentrations and decreased at high salt concentrations. Parachinar-local soybean accumulated much protein in comparison to swat-84, indicating its high ability to tolerate salt stress. Studies have shown that jasmonates induce stress proteins that assist plants in coping with stress conditions and regulating important processes of plant growth [52,70,71,72]. In our study, exogenous JA application increased the phenol concentration in both soybean varieties, compared with that in the control. It has been reported that JA can act as a signaling molecule for signal transduction and induce gene expression levels of phenylalanine ammonia-lyase (PAL), which catalyzes the biosynthesis of phenolic compounds [62]. Phenolic compounds possess antioxidant properties, scavenging free radicals and helping plants cope with stress [73].

Further, we found that vitamin A concentration decreased under medium and high salt stress but increased under low salt stress. However, exogenous JA application alone and in combination with salt stress increased vitamin A concentration in both soybean varieties. The JA has been demonstrated to be involved in the activation or biosynthesis of certain transcription factors responsible for the amelioration of vitamins [52,74]. A plethora of studies have demonstrated that exogenous JA triggers the anti-oxidant system and alleviates the damage associated with stress conditions [23,52,75]. Therefore, a high concentration of phenol and vitamin A accumulation in parachinar-local soybean might explain its better growth performance compared with that in the swat-84 soybean.

## 4. Materials and Methods

### 4.1. Experimental Setup 

A randomized complete block design experiment (RCBD) pot experiment was conducted in the greenhouse of the Department of Botany, Islamia College University, Peshawar (latitude, 34.025917 “N, longitude, 71.560135 E, and altitude, 331 m), during the soybean growing season of 2018. The seeds (Parachinar-local and Swat-84) were generously provided by the Cereal Crop Research Institute (CCRI), Nowshera, and Nuclear Institute for Food and Agriculture (NIFA), Peshawar, Pakistan. Seeds (95% viability rate) sterilized with 5% Clorox, washed thrice with 50% ethanol, rinsed with distilled water, and then sown in a total of 48 plastic pots (18.5 cm upper inner diameter, 14 cm lower inside diameter, 0.5 cm thickness, and 15.6 cm height) filled with air-dried soil and sand (3:1) in triplicates. All the pots were placed in an incubator with average day and night temperatures of 25 °C (10 h) and 16 °C (14 h), respectively, and were watered as required. Two-week-old seedlings were divided into four sets: (a) distilled water, (b) distilled water + JA, (c) NaCl stress, and (d) NaCl stress + JA. Four different concentrations of NaCl stress (0, 40, 60, 120 mM) were applied to the surfaces of the pots. Further, exogenous JA solution (100 μmol L^−1^) was sprayed on the upper surface of the leaves on the same day as salt stress treatments. There were seven treatments of both salt and exogenous JA foliar spray. Moreover, there was a total of five plants per pot, and three replicates were used for each treatment.

### 4.2. Growth Parameters

Following the harvest of the soybean seedlings, the shoot height, root length, shoot and root fresh weight, and shoot and root dry weight were measured [76]. 

### 4.3. Determination of Photosynthetic Pigments 

A solution of 80% acetone and anhydrous ethanol (1:1) was used to extract photosynthetic pigments completely from dry leaf samples (0.1 g). A spectrophotometer was used to measure absorbance at 440 nm (Car), 645 nm (Chl a), and 663 nm (Chl b), and their concentrations (mg g^−1^) were calculated according to standard protocol [77]. 

### 4.4. Determination of Foliar Mineral Nutrients Concentration

Two plants were harvested and oven-dried (70 °C) for the determination of NO_3_^−^, Cl^−^, SO_4_^2−^, and H_2_PO^4−^ contents using ion chromatography. In addition, an atomic absorption spectrophotometer was used to determine the contents of Na^+^, K^+^, Fe^2+^, Mg^2+^, Mn^2+^, Zn^2+^, B^3+^, and P^3+^ ions [78].

### 4.5. Determination of Foliar Sugar, Protein, Total Phenol, and Vitamin A 

For further analysis, fresh leaves from the rest of the plants were harvested, cut, washed with distilled water, immediately immersed in liquid nitrogen, and then stored at -80 °C. Moreover, the sugar content was estimated following the standard method of [79]. The absorbance was recorded at 420 nm. The standard curve was made using glucose to calculate the concentration. Protein content was determined following the standard protocol, [80], using BSA as standard. Total phenol and vitamin A content were determined following the methods of [81] and [82], respectively. 

### 4.6. Statistical Analysis

Analysis of variance (ANOVA) was performed in SPSS (v.13.0; IBM, Armonk, NY, USA), and significant differences among treatments mean were detected using Duncan’s test at *p* < 0.05. GraphPad Prism 8.0.1 was used to draw the figure graphics, all of which show data points and error bars as the mean ± SE.

## 5. Conclusions

In conclusion, exogenous jasmonic acid (JA) application alleviated the negative impact of salt stress in terms of the growth and metabolism of both soybean varieties. The swat-84 exhibited a greater salt-induced growth and metabolism reduction than the parachinar-local variety. Further, the protein and phenol content of soybean seedlings may have a significant impact on the interplay among their multiple attributes. Therefore, further studies on the accumulation of proteins and phenol in young seedlings treated with exogenous JA foliar spray may reveal important information regarding plant growth strategies under salt stress. Further, it is necessary to gain a greater understanding of the molecular mechanism behind the ameliorative effects of exogenous JA application on the growth and metabolism of both soybean varieties under salinity conditions.

## Figures and Tables

**Table 1:** Changes in shoot growth of both soybeans.

Soybean Variety	Treatments	Shoot Height (cm)	Shoot Fresh Weight (g)	Shoot Dry Weight (g)	Shoot Moisture Content
Parachinar-local	T1	29.00 ± 5.27 ab	7.79 ± 0.27 abc	2.93 ± 1.25 ab	4.86 ± 1.14 a
	T2	32.67 ± 2.95 a	13.88 ± 4.50 a	3.59 ± 0.89 a	10.29 ± 3.62 a
	T3	31.43 ± 4.46 ab	14.00 ± 2.58 a	2.83 ± 0.54 ab	11.17 ± 3.09 a
	T4	30.67 ± 2.49 ab	14.17 ± 1.83 a	3.64 ± 0.28 a	10.53 ± 1.55 a
	T5	22.00 ± 1.15 ab	6.03 ± 1.90 bc	1.37 ± 0.46 b	4.66 ± 1.46 a
	T6	27.90 ± 0.49 ab	12.97 ± 2.52 ab	2.34 ± 0.29 ab	10.63 ± 2.81 a
	T7	21.40 ± 1.70 b	5.35 ± 0.62 c	1.39 ± 0.51 b	3.96 ± 1.08 a
	T8	27.03 ± 4.23 ab	11.46 ± 1.42 abc	2.57 ± 0.13 ab	8.89 ± 1.37 a
Swat-84	T1	35.10 ± 0.95 a	17.60 ± 2.92 a	4.82 ± 0.55 a	12.77 ± 2.37 a
	T2	31.07 ± 2.10 ab	13.90 ± 3.18 ab	3.56 ± 0.76 ab	10.33 ± 2.42 ab
	T3	27.70 ± 0.42 bc	9.56 ± 2.75 ab	2.35 ± 0.73 ab	7.21 ± 2.03 ab
	T4	30.33 ± 2.42 bc	11.33 ± 2.32 ab	3.04 ± 0.86 ab	8.29 ± 2.75 ab
	T5	29.13 ± 0.52 bc	11.08 ± 3.55 ab	2.50 ± 0.76 ab	8.58 ± 2.79 ab
	T6	26.33 ± 0.88 cd	11.44 ± 0.86 ab	2.98 ± 0.52 ab	8.46 ± 0.34 ab
	T7	23.47 ± 0.69 d	6.55 ± 0.70 b	2.09 ± 0.21 b	4.46 ± 0.63 a
	T8	31.50 ± 0.75 ab	16.43 ± 4.27 a	4.50 ± 1.14 ab	11.93 ± 3.22 ab

The values are shown as mean ± SE. Different letters for each column for each variety show significantly different values at *p* < 0.05 according to Duncan’s method. T1 (Control), T2 (Control + JA), T3 (40 mM), T4 (40 mM + JA), T5 (80 mM), T6 (80 mM + JA), T7 (120 mM), T8 (120 mM + JA).

**Table 2:** Changes in root growth of both soybeans.

Soybean Variety	Treatments	Root Length (cm)	Root Fresh Weight(g)	Root Dry Weight (g)	Root Moisture (g)
Parachinar-local	T1	20.67 ± 0.67 ab	1.19 ± 0.18 abc	0.52 ± 0.10 a	0.67 ± 0.08 abc
	T2	16.60 ± 3.15 b	1.31 ± 0.31 abc	0.65 ± 0.14 a	0.66 ± 0.18 abc
	T3	17.43 ± 1.34 ab	2.11 ± 0.44 a	0.71 ± 0.26 a	1.40 ± 0.18 ab
	T4	19.33 ± 3.38 ab	1.81 ± 0.64 ab	0.67 ± 0.05 a	1.13 ± 0.59 ab
	T5	17.03 ± 3.07 b	0.79 ± 0.21 bc	0.35 ± 0.11 a	0.45 ± 0.10 bc
	T6	24.43 ± 1.84 a	1.84 ± 0.46 ab	0.36 ± 0.08 a	1.48 ± 0.38 a
	T7	16.33 ± 1.20 b	0.61 ± 0.06 c	0.48 ± 0.01 a	0.12 ± 0.06 c
	T8	22.67 ± 1.01 ab	1.30 ± 0.14 abc	0.49 ± 0.12 a	0.81 ± 0.26 abc
Swat-84	T1	26.23 ± 0.77 a	1.80 ± 0.30 a	0.78 ± 0.10 a	1.03 ± 0.21 a
	T2	14.33 ± 1.20 cd	1.13 ± 0.29 a	0.51 ± 0.11 a	0.62 ± 0.19 a
	T3	17.00 ± 1.53 bcd	0.63 ± 0.17 a	0.38 ± 0.08 a	0.24 ± 0.10 a
	T4	22.67 ± 5.21 ab	1.14 ± 0.26 a	0.44 ± 0.13 a	0.70 ± 0.23 a
	T5	19.77 ± 0.83 abc	1.52 ± 0.61 a	0.43 ± 0.15 a	1.09 ± 0.49 a
	T6	19.30 ± 0.69 abc	1.47 ± 0.31 a	0.67 ± 0.17 a	0.80 ± 0.15a
	T7	12.03 ± 1.74 d	1.01 ± 0.27 a	0.45 ± 0.08 a	0.56 ± 0.20 a
	T8	24.00 ± 1.15 ab	1.66 ± 0.63 a	0.80 ± 0.29 a	0.86 ± 0.34 a

The values are shown as mean ± SE. Different letters for each column for each variety show significantly different values at *p* < 0.05 according to Duncan’s method. T1 (Control), T2 (Control + JA), T3 (40 mM), T4 (40 mM + JA), T5 (80 mM), T6 (80 mM + JA), T7 (120 mM), T8 (120 mM + JA).

**Table 3:** Changes in foliar anions concentration of both soybeans.

Soybean Variety	Treatments	NO_3_^−^	Cl^−^	SO_4_^2−^	H_2_PO_4_^−^
Parachinar-local	T1	10.26 ± 0.84 a	8.61 ± 0.54 d	17.46 ± 0.51 f	10.87 ± 0.47 a
	T2	9.06 ± 0.70 ab	6.06 ± 0.30 c	23.69 ± 2.91d e	10.45 ± 0.55 a
	T3	6.92 ± 0.23 c	12.85 ± 0.42 bc	22.58 ± 2.74 ef	9.82 ± 0.51 ab
	T4	7.98 ± 0.37 bc	12.21 ± 0.55 b	21.56 ± 2.42 ef	10.16 ± 0.58 ab
	T5	6.58 ± 0.23 cd	14.29 ± 0.15 a	28.93 ± 0.98 cd	8.84 ± 0.34 bc
	T6	7.41 ± 0.41 c	12.15 ± 1.07 b	31.85 ± 0.69 bc	9.81 ± 0.39 ab
	T7	5.15 ± 0.31 d	14.11 ± 0.15 a	36.08 ± 0.58 ab	7.44 ± 0.30 d
	T8	5.37 ± 0.29 d	12.27 ± 0.27 b	37.46 ± 1.12 a	7.83 ± 0.19 cd
Swat-84	T1	7.27 ± 0.17 a	8.88 ± 0.88 e	17.32 ± 1.04 g	10.60 ± 0.53 a
	T2	6.62 ± 0.31 b	6.47 ± 0.36 f	24.39 ± 0.12 e	9.70 ± 0.48 ab
	T3	5.37 ± 0.31 c	14.69 ± 0.70 d	20.69 ± 2.00 f	9.13 ± 0.49 abc
	T4	5.99 ± 0.23 c	15.35 ± 0.58 cd	24.72 ± 0.75 de	9.46 ± 0.50 abc
	T5	4.29 ± 0.15 d	17.29 ± 0.46 b	29.32 ± 1.77 bc	8.77 ± 0.42 bcd
	T6	4.73 ± 0.18 d	16.74 ± 0.51 bc	27.94 ± 0.40 cd	8.97 ± 0.48 bcd
	T7	3.03 ± 0.04 e	19.33 ± 0.28 a	31.72 ± 0.48 b	7.48 ± 0.56 d
	T8	3.36 ± 0.14 e	18.03 ± 0.47 ab	36.97 ± 0.97 a	7.70 ± 0.48 cd

The values are shown as mean ± SE. Different letters for each column for each variety show significantly different values at *p* < 0.05 according to Duncan’s method. T1 (Control), T2 (Control + JA), T3 (40 mM), T4 (40 mM + JA), T5 (80 mM), T6 (80 mM + JA), T7 (120 mM), T8 (120 mM + JA).

**Table 4:** Changes in foliar cations concentration of both soybeans.

Soybean Variety	Treatments	Na^+^	K^+^	Mg^2+^	P^3+^
Parachinar-local	T1	20.36 ± 0.79 d	177.98 ± 3.85 a	42.14 ± 2.50 a	21.19 ± 1.45 b
	T2	18.21 ± 0.67 cd	167.31 ± 5.84 ab	34.85 ± 1.29 b	24.61 ± 1.37 a
	T3	22.71 ± 0.54 cd	161.48 ± 5.01 bc	29.38 ± 0.77 cd	20.82 ± 1.02 b
	T4	20.93 ± 1.00 cd	152.94 ± 3.69 cd	31.42 ± 0.97 bcd	22.48 ± 1.22 ab
	T5	29.93 ± 1.10 b	140.76 ± 6.59 de	33.59 ± 1.73 bc	16.15 ± 1.13 c
	T6	22.90 ± 1.13 c	128.66 ± 3.07 ef	34.04 ± 1.04 b	14.35 ± 0.39 cd
	T7	33.04 ± 0.97 a	118.92 ± 3.88 f	28.04 ± 0.66 d	12.48 ± 1.18 d
	T8	28.66 ± 0.68 b	128.53 ± 2.92 ef	30.68 ± 0.85b cd	11.78 ± 0.26 d
Swat-84	T1	22.56 ± 1.23 d	163.02 ± 6.47 a	41.64 ± 2.55 a	14.06 ± 0.40 a
	T2	19.51 ± 0.38 d	139.57 ± 8.57 b	37.57 ± 0.92 ab	15.52 ± 0.87 a
	T3	36.08 ± 1.33 c	141.49 ± 5.45 b	33.84 ± 1.04 bc	13.91 ± 0.61 a
	T4	34.59 ± 1.09 c	91.63 ± 5.91 d	35.69 ± 0.34 b	13.13 ± 0.61 a
	T5	40.02 ± 1.09 b	120.26 ± 3.12 c	29.11 ± 1.49 de	9.64 ± 0.66 a
	T6	32.58 ± 1.13 c	85.60 ± 7.18d e	30.97 ± 2.07 cd	10.52 ± 1.05 a
	T7	48.59 ± 2.02 a	65.60 ± 4.20 f	24.25 ± 0.42 f	10.12 ± 0.59 a
	T8	42.64 ± 1.63 b	69.20 ± 2.12 ef	25.26 ± 1.03 ef	10.19 ± 1.27 a

The values are shown as mean ± SE. Different letters for each column for each variety show significantly different values at *p* < 0.05 according to Duncan’s method. T1 (Control), T2 (Control + JA), T3 (40 mM), T4 (40 mM + JA), T5 (80 mM), T6 (80 mM + JA), T7 (120 mM), T8 (120 mM + JA).

**Table 5:** Changes in foliar cations concentration of both soybeans.

Soybean Variety	Treatments	Mn^2+^	B^3+^	Zn^3+^	Fe^3+^
Parachinar-local	T1	0.08 ± 0.00 a	0.34 ± 0.03 b	0.08 ± 0.00 a	1.44 ± 0.10 a
	T2	0.10 ± 0.01 a	0.43 ± 0.02 a	0.08 ± 0.00 ab	1.60 ± 0.16 a
	T3	0.07 ± 0.00 a	0.31 ± 0.02 bc	0.07 ± 0.00 cd	1.47 ± 0.09 a
	T4	0.10 ± 0.01 a	0.32 ± 0.01 bc	0.07 ± 0.00 bc	1.70 ± 0.14 a
	T5	0.06 ± 0.01 a	0.27 ± 0.03 cde	0.05 ± 0.00 ef	1.72 ± 0.32 a
	T6	0.10 ± 0.03 a	0.28 ± 0.01b cd	0.06 ± 0.00 de	2.06 ± 0.43 a
	T7	0.06 ± 0.01 a	0.22 ± 0.02 d	0.04 ± 0.00 f	1.96 ± 0.36 a
	T8	0.07 ± 0.00 a	0.24 ± 0.01 de	0.05 ± 0.00 ef	2.26 ± 0.43 a
Swat-84	T1	0.14 ± 0.01 a	0.48 ± 0.01 ab	0.09 ± 0.01 a	1.48 ± 0.18 a
	T2	0.14 ± 0.00 a	0.52 ± 0.08 a	0.08 ± 0.00 ab	1.70 ± 0.43 a
	T3	0.09 ± 0.00 cd	0.37 ± 0.01 c	0.06 ± 0.00 bcd	1.80 ± 0.39 a
	T4	0.10 ± 0.01 bc	0.39 ± 0.01 bc	0.07 ± 0.01 bc	1.64 ± 0.27 a
	T5	0.11 ± 0.00 b	0.32 ± 0.01 c	0.05 ± 0.00 cd	1.50 ± 0.19 a
	T6	0.11 ± 0.00 b	0.35 ± 0.02 c	0.06 ± 0.00 bcd	1.65 ± 0.26 a
	T7	0.08 ± 0.00 d	0.29 ± 0.01 c	0.05 ± 0.01 d	1.59 ± 0.41 a
	T8	0.09 ± 0.00 cd	0.30 ± 0.01 c	0.05 ± 0.01 cd	1.80 ± 0.43 a

The values are shown as mean ± SE. Different letters for each column for each variety show significantly different values at *p* < 0.05 according to Duncan’s method. T1 (Control), T2 (Control + JA), T3 (40 mM), T4 (40 mM + JA), T5 (80 mM), T6 (80 mM + JA), T7 (120 mM), T8 (120 mM + JA).

## Data Availability

Not applicable.
